# Bacteriological quality and associated risk factors of drinking water in Eastern zone, Tigrai, Ethiopia, 2019

**DOI:** 10.1186/s40794-020-00116-0

**Published:** 2020-08-28

**Authors:** Aderajew Gebrewahd, Gebre Adhanom, Gebremedhin Gebremichail, Tsega Kahsay, Brhane Berhe, Zinabu Asfaw, Senait Tadesse, Haftay Gebremedhin, Hadush Negash, Brhane Tesfanchal, Hagos Haileselasie, Haftom Legese Weldetinsaa

**Affiliations:** 1 Department of Medical Laboratory, College of Medicine and Health Science, Adigrat University, Adigrat, Ethiopia; 2 Department of public health, College of Medicine and Health Science, Adigrat University, Adigrat, Ethiopia; 3 Department of Medical Laboratory, College of Medicine and Health Science, Bahir Dar University, Bahir Dar, Ethiopia

**Keywords:** Bacteriological quality, Ethiopia, Faecal coliforms, Tigrai, Total coliforms, Water sources

## Abstract

**Background:**

Access to safe drinking water is one of the basic human rights and is critical to health. However, much of the world’s population lacks access to adequate and safe water. Approximately 884,000, 000 people in the world still do not get their drinking water from safe sources; Sub-Saharan Africa accounts for over one third of this number. It is estimated that 80% of all illnesses in the world are related to use of unsafe and contaminated water.

**Methods:**

A cross-sectional study was conducted from August 1st 2017 to July 30th 2018 in three randomly selected woreda (districts) of Eastern Zone Tigrai. Water samples were examined for total coliforms and thermotelorant coliforms using the most probable number method. Standard biochemical testing was performed on samples that tested positive to identify the genus of bacteria. The contaminant risk of water sources were assessed using the sanitary inspection checklist of the World Health Organization. The results were interpreted using World Health Organization guidelines for drinking water quality**.** Data was collected using laboratory checklist and sanitary inspection check list. It was entered, cleared and analyzed using SPSS version 21.0 and a variable having a *P* < 0.05 was considered as statistically significant in all tests.

**Results:**

A total of 290 drinking water samples were analyzed for bacteriological quality. A total of 32.4% (*n* = 94) of water sources showed contamination with faecal and total coliforms. Of these 3.4% (*n* = 10) samples were contaminated with total coliforms and 29% (*n* = 84) contaminated with faecal coliforms. The leading water contaminant organisms were *Escherichia coli* (62.4%), *Legionella* species (8.5%), and *Shigella* species (7.5%) respectively. Based on WHO criteria, 15% of water sources were grouped in the very high risk group. Animal excreta and inadequate fencing of water sources were significantly associated with water contamination rate.

**Conclusion:**

Our findings suggest that most water sources in woredas of Eastern Tigrai are contaminated by faecal coliforms. Therefore, regular sanitary inspection, bacteriological analysis, and adequate fencing should be mandatory to protect drinking water sources from faecal contamination.

## Background

Bacteriological water quality is defined in terms of the absence or presence of indicator organisms. Drinking water does not cause an infectious disease if it is free from indicator organisms [1].

Access to safe drinking water is one of the basic human rights and is extremely important for health. For a country to maintain optimal health and development there has to be a continuous supply of safe drinking water to its population [2]. However, most of the world’s population lacks access to adequate and safe water [3]. Eight hundred and eighty four million people in the world do not have access to safe drinking water. Sub-Saharan Africa accounts for over one third of this number **[**4**]**.

About two thirds of drinking water consumed worldwide is derived from surface water sources such as lakes, rivers and open wells. Therefore, it can be easily contaminated with micro-organisms from sewage discharge or human and animal feces [5]. Contaminated water serves as a mechanism to transmit communicable diseases such as cholera, dysentery, typhoid, and parasitic infections [1]. As a result, waterborne diseases continue to be one of the major health problems worldwide [6]. It is estimated that 80% of all illnesses in the world can be traced back to contaminated drinking water [7].

About 1.8 million deaths per year are attributed to unsafe water supply due to inadequate sanitation and hygiene. Children under five are the most affected group [8]. However, improvement in water supply can help to reduce overall morbidity by 6 to 25% [9].

Inadequate access to safe water and sanitation services negatively impacts the health and economy of a country [10]. Infectious diseases caused by pathogenic microorganisms are the most common and widespread health risk associated with drinking water [1]. The greatest risk to public health from microbes in water is associated with consumption of drinking water that is contaminated with human and animal excreta. Every year, more people die from waterborne illnesses than all forms of violence, including war [11].

Waterborne pathogens cause diseases in approximately 250 million people each year, resulting in 10 to 20 million deaths around the globe [12]. These pathogens continue to occur as outbreaks [13] and contribute to 80% of health problems in developing countries [14].

Ethiopia is one of the developing countries where only 57 and 28% of its population have access to safe water and sanitation coverage respectively. A total of 60–80% of the population suffers from waterborne and water-related diseases. This places a significant financial and social burden on the country with such a large number of people suffering from these devastating diseases [15]. School absenteeism in children and loss of productivity due to illness are common consequences of waterborne diseases.

Reports have shown that from underground water sources (hand-dug wells and springs) in rural parts of North West Ethiopia and hand-pumped wells in Shashemene District, West Arsi zone of Oromia region, 63.6 and 91.6% of the water samples were positive for total coliforms and thermo-tolerant coliforms respectively [16, 17].

Simply improving the quality of drinking water source may not solve the problem because people can become infected with micro-organisms through many other ways [18]. Therefore, in addition to water improvements at the source (e.g. protected wells, hand-pump, spring and tap stands), improvements in hygiene and sanitation practices are also important to minimize the risk of waterborne diseases [19].

Government regulations and research has centered on microbial risk assessment and management in the water sector; however, application and interpretation of findings has been lacking [20]. Therefore, the aim of this study was to assess the bacteriological quality and associated risk factors of drinking water in Eastern zone, Tigrai, Ethiopia.

## Materials and methods

### Description of the study area

The study was conducted in Eastern Zone of Tigrai region. Eastern Zone is bordered with Afar region in east, Southern East Tigrai in the south, Central Zone Tigrai in the west, and Eritrea in the north. The Zone has seven woredas and two town administrations (Adigrat and Wukro). The Eastern Zone administration town, Adigrat is located 903 km from Addis Ababa and 120 km from Mekelle (the Tigrai regional capital), with latitude of 14^0^ 16ʼ north and longitude of 39^0^ 27′ east, and an elevation of 2457 m (8061 ft) above sea level (https://en.wikipedia.org/wiki/Misraqawi).

Eastern zone has an estimated area of 4717.5 km^2^ and based on the 2016 census conducted by the central statistical agency of Ethiopia, has a total population of 900,515. Of these, 428,952 are males and 471,563 are females (Eastern zone administration).

### Sample size determination

The sample size was calculated by using the single population proportion formula.

$$ \mathrm{n}=\frac{{\mathrm{z}}^2\upalpha /2\mathrm{p}\left(1\hbox{-} \mathrm{p}\right)}{{\mathrm{d}}^2} $$

$$ \mathrm{n}=\frac{(1.96)^2(0.9)(0.1)}{(0.05)^2}=138 $$

By assuming: z = 95% confidence interval i.e.1.96 p = previous proportion of contaminated water i.e. 0.9 [4], d = margin of error (0.05), n = by adding 5% no functionality rate of water sources and multiplied with design effect of 2, the calculated total sample size was 290.

### Sampling techniques

A multi-stage stratified sampling method was used to select water sources from each kebele (neighborhood) for the study. Three woredas were selected from the total of nine woredas of Eastern zone using random sampling technique. These were Ganta Afeshum, Gulomekeda and Saesiet Tsaeda emba woredas. The three woredas had 63 kebeles, and out of these, 21 kebeles were included in this study by random sampling. The kebeles (https://en.wikipedia.org/wiki/Misraqawi) had a total of 460 functional water sources and the calculated sample size was proportionally allocated to the randomly selected kebeles based on the available functional water sources.

### Sample collection

Water samples were collected according to the WHO Guidelines for drinking water quality assessment (drinking water must be collected aseptically using a sterile bottle and bacteriological analysis should be performed within 6 h of sample collection) [21]. The samples were collected from hand pumps (264 samples) and springs (26 samples) by trained data collectors that are used directly for drinking purposes in the community. When collecting the sample, the interior or mouth of the container and container caps were never touched with fingers, clothing, or unsterile objects. Before taking samples from hand pumps, the hand pump water was flushed for 2 min and then the mouth of the hand pump was sterilized with a spirit of lamp flame and then cooled by running water [22]. Water (200 ml) samples from water sources were collected in 300 ml glass bottles (sterilized by hot air oven at 160 °C for 2 h) and transported within 30 min in a cold box to Adigrat University Department of Medical Laboratory Sciences skill laboratory. Samples containing residual chlorine were neutralized by adding 0.2 ml sodium thiosulphate per 200 ml of water sample. The samples were stored at 2 °C–8 °C in a dark area to avoid changes in microbial count until analysis. Microbiological investigations were performed within 6 h after collection [23]. In addition to that, the contaminant risks of water sources were assessed using the WHO sanitary observation check list for drinking water sources [24]. The sanitary observation check list was used to assess potential risk factors for drinking water sources.

### Bacteriological water analysis

Presumptive coliform test was performed using MacConkey broth (Oxoid) [25]. All the tubes contained Durham tubes before sterilization. The first set of three tubes had 10 ml of sterile double strength broth and the second and the third sets had 10 ml of single strength broth. The three sets of tubes would receive 10 ml, 1 ml and 0.1 ml quantities of water samples using sterile syringes respectively and were incubated for 48 h at 37 °C. Tubes showing gas formation were considered presumptive coliform positive. The most probable number (MPN) was then estimated from the table for three tube test [22].

Confirmatory testing was carried out by transferring a loop full of culture from each tube which showed acid and gas in the presumptive test and inoculating it in to Brilliant Green Lactose Bile (BGLB) broth (Oxoid) and *Escherichia coli* (*E. coli*) medium. The inoculated tubes were incubated at 37 °C for 48 h for total coliforms and 44.5 °C for 24 h for thermotolerant coliforms in water bath. Then, after 24–48 h of incubation, samples were monitored for gas production and standard biochemical testing was performed to identify water contaminants up to genus level [25].

### Dataquality assurance

Data collectors and supervisors were trained with regular supervision by the principal investigator. Prior to the actual work, reagents were checked for proper functioning and sterility of the prepared culture media was checked by incubating 5% representative of the batch culture at 37 °C overnight and observing for bacterial growth.

A standard strain of *Escherichia coli* ATCC 25922 was used during culturing as a quality control. The collected data was reviewed and checked for completeness and consistency by the supervisors and principal investigator each day. The data was checked, coded, and entered into a computer before analysis**.**

### Data processing and analysis

Data was entered and analyzed using Statistical Product and Service Solutions (SPSS) version 21 and frequencies and percentages were used to present categorical data. Odds ratios (ORs), 95% confidence intervals (CIs), and *P*-values were calculated using a logistic regression model to determine association levels of independent variables to the dependent variables. Crude ORs of independent variables with bacteriological quality of water sources were estimated using bivariate logistic regression analysis. Multivariate logistic regression analysis was used to estimate the adjusted OR of independent variables to bacteriological quality of water by controlling confounding factors. A variable having *P* < 0.05 was considered as statistically significant.

## Results

In this study, a total of 290 drinking water samples were analyzed for bacteriological quality. A total of 32.4% (*n* = 94) of the water sources showed contamination (Table 1). A total of 3.4% (*n* = 10) of samples were contaminated with total coliforms and 29% (*n* = 84) were contaminated with faecal coliforms (Table 1). Total coliform bacteria are the type of microorganisms that can be found in the aquatic environment, soil, and vegetations. Faecal coliforms are the coliform bacteria that originated specifically from the intestinal tracts of warm- blooded animals and can grow at 44.5 °C within 48 h of incubation. The remaining of 67.6% (*n* = 196) water samples were bacteriologically potable.

**Table 1:** Bacteriological water potability and isolated indicator organisms with water source type from the analyzed water samples in Eastern zone Tigrai, Ethiopia, 2019

Water source type	Bacteriological potability			Isolated indicator organism		
	potable	Non potable	Total	Faecal coliform	Total coliform	Total
Hand pump	196	68	264	58	10	68
spring	0	26	26	26	0	0
total	196	94	290	84	10	94

Water samples were collected from two drinking water sources and the microbiological analysis of these sample showed that the lowest contamination was in hand-pump water sources while the highest contamination was shown in spring water sources.

A total of 26% (*n* = 68) of hand-pump water sources and 100% (*n* = 26) of spring water sources were contaminated with bacteria agents.

### Distribution of identified organisms among water sources types

From the contaminated water samples, 62.8% (*n* = 59), 8.5% (n = 8), and 7.5% (n = 7) were positive for *Escherichia coli*, *Legionella,* and *Shigella* respectively. Among the total positive water samples, *Escherichia coli* were found in 59.6% (*n* = 56), *Pseudomonas* species in 7.5% (*n* = 7), and *Legionella* species in 4.3% (n = 4) of hand pump water sources. Likewise, among the total positive water samples again *Shigella* was detected in 7.5% (n = 7), *Yersinia* species in 7.5% (n = 7), and *Legionella* in 4.3% (*n* = 4) of the spring water sources (Table 2).

**Table 2:** Distribution of isolated bacteria with water source types recovered from positive water samples of Eastern zone Tigrai, Ethiopia, 2019

Water source	***E.coli***	***Salmonella*** species	***Shigella*** species	***Yersinia***	***Campylobacter***	***Legionella***	***Pseudomonas***	Total
**Hand pump**	56(59.6%)	0	0	0	1(1%)	4(4.3%)	7(7.5%)	68(72.3%)
**Spring**	3(3.2%)	3(3.2%)	7(7.5%)	7(7.5%)	1(1%)	4(4.3%)	1(1%)	26(27.7%)
**Total**	59(62.8%)	3(3.2%)	7(7.5%)	7(7.5%)	2(2%)	8(8.5%)	8(8.5%)	94(100)

**Note:** ***E. coli*-** ***Escherichia coli***

### Sanitary inspection results

A sanitary risk score was computed as qualitative risk category (low, medium, high, and very high risks) for each water source by putting the number of positive factors as a range (0–2, 3–5, 6–8 and 9–10) of the total number of factors being assessed. According to the result obtained from the sanitary inspection of the assessed water sources (Table 3), only 15% of the water sources were grouped into the” very high” contamination risk category, whereas the percentage of the water sources clustered into” Low”,” Intermediate,” and” High” contamination risk category were 31, 20, and 34% respectively (Table 3).

**Table 3:** Contamination risk level percentage of the assessed water sources

Sanitation score	Frequency	Percent	Risk category
**0–2**	29	31	Low
**3–5**	19	20	Medium
**6–8**	32	34	High
**9–10**	14	15	Very high
**Total**	94	100	

### Association of risk factors with bacterial contaminants of the assessed water sources

Out of the total drinking-water sources found positive for total coliforms and faecal coliform bacteria, 21.6% (*n* = 57) had animal excreta around the source, 34.1% (*n* = 90) had inadequate fences, and 10.6% (*n* = 28) had permitted ponding (allowing for accumulation of) water around the concrete floor. The presence of animal excreta (COR9.600, 95% CI: 6.50–79.81) and inadequate fencing (COR8.200, 95% CI: 1.53–258.24) around water sources were significantly associated with bacterial contamination of drinking water from the hand pump. The association was not statistically significant with concrete floor < 1 m around the hand pump in the adjusted odds ratio analysis (AOR1.23, 95% CI: 0.09–16.12). The unfenced area around the spring was significantly correlated with the contamination rate of drinking water from spring sources in the binary model analysis. Absence of fencing around the spring was significantly associated with higher contamination on binary model analysis (COR6.37, 95% CI: 1.82–83.76), but it was not statistically significant in the adjusted odds ratio (AOR 4.28, 95% CI: 0.43–42.76). The presence of cracking and ponding on the concrete floor and unsanitary cover of the hand-pump water sources were not statistically associated with the rate of water bacterial contaminations (Table 4).

**Table 4:** Logistic regression models of faecal and total coliform bacteria presence in the assessed water sources of Eastern zone Tigrai, Ethiopia, 2019

Variables		COR(95%CI)	***p***-value	AOR(95%CI)	***p***-value
**Animal excreta around hand- pump**	Yes 57(21.6%)	9.60(54.020–532.475	0.001	7.78(6.506–79.810)	0.001*
	No 207(78.4%)			1	
**Ponding on the concrete floor around the hand- pump**	Yes 28(10.6%)	1.17(0.491–2.803)	0.719		
	No 236((89.4%)			1	
**Inadequate fencing allowing animal access to hand- pump**	Yes 90(34.1%)	8.20(43.268–507.605)	0.007	6.41(8.419–124.372)	0.003*
	No174(65.9)			1	
**Cracks permit water to enter the hand- pump**	Yes 3(1.1%)	5.90(0.527–66.229)	0.150		
	No 261(98.9%)			1	
**Concrete floor < 1 m around the hand- pump**	Yes 8(3%)	2.37(2.700–185.483)	0.004	1.23(0.095–16.125)	0.872
	No 256(97%)			1	
**Cover of hand- pump unsanitary**	Yes 24(9.1%)	0.38(0.111–1.333)	0.132		
	No 240(90.9%)			1	

Note: *- statistically significant; *COR* crude odds ratio; *AOR* adjusted odds ratio; *95%CI* 95% confidence intervals; 1- referent

## Discussion

The World Health Organization recommends that water directly used for human consumption should be free from microbial contamination, since the presence of *Escherichia coli* indicates a potential health risk for consumers [1].

However, the current study showed that from the total 264 collected hand pump water samples, 56 (21.2%) of them were contaminated with *Escherichia coli*. On the other hand, from the total 26 collected spring water samples, 3 (11.5%) of them had *Escherichia coli* concentration above the WHO acceptable range for drinking water. The same study in North Gonder zone showed that 35.7% of the samples obtained from protected springs had *Escherichia coli* [26].

Another study in rural communities of Dire Dawa administrative council showed that all protected springs were positive for total coliforms [27]. Similarly, in the present study, all spring water sources were positive for faecal coliforms, but none of them were contaminated with total coliforms. As faecal coliform bacteria are found only in human/animal intestinal tracts, their presence in drinking water indicated that it may most likely be contaminated with gastrointestinal pathogenic bacteria. This suggested that, poor protection and sanitation practice in the water sources handling practices may be the reason for contamination with faecal coliforms.

In our study, from the total 290 analyzed water samples, there were 196 water sources with MPN result of zero per 100 ml of water. All of them were from hand pumps whereas there was no water source with MPN zero per 100 ml from unprotected spring water sources, showing that hand pump water sources are safer than unprotected spring sources due to the availability of concrete covers in hand pump water sources restricting the entry of animal and human excreta.

In the present study, *Escherichia coli* (20.3%) was the predominant pathogen isolated from the drinking water sample, with the second and third most isolated being *Legionella* and *Pseudomonas* species (2.8%). Similar studies in Sudan, Darfur (22.5%), and Nigeria (33%) also reported that the predominant drinking water contaminant was *Escherichia coli*. However, in the studies conducted in Nigeria and Sudan, Darfur, the second and third most bacterial isolated were *Klebsiella* species (17%) and *Enterococcus faecalis* (20.42%) respectively rather than *Legionella* species [28, 29]. This may be due to geographical differences of the organisms and sources of water contaminants. In contrast to our finding, another study conducted in Ethiopia, Tigrai region also showed that *Klebsiella* species was the predominant pathogen isolated [30].

The present study had sanitary risk scores ranging from low to high. Around 34% of water sources had a high sanitary risk score for coliform contamination. This finding is in agreement with a study conducted in rural communities of Ethiopia [16, 31]. They reported that all dug wells and springs were at high risk category for total coliforms. Another study in Sidama Zone, Bona district showed that all of the protected spring examined had risks ranging from low to high [32].

The presence of animal excreta and inadequate fencing around water sources were significantly associated with bacterial contamination of drinking water. This may be due to contamination of water sources with animal’s excreta especially for those without fences.

### Limitations

As a limitation, the physiochemical analysis and other water contaminant organisms (virus, parasite and fungi) of water was not done due to financial constraints.

## Conclusion

In conclusion, 32.4% of the water sources had unacceptable faecal and total coliform count. The predominant isolated organism was *Escherichia coli.* All the water sources which were positive for bacterial coliform count had *Escherichia coli* showing faecal contamination of water sources. Therefore, we recommend regular disinfection, bacteriological examination and inspection of drinking water sources. Furthermore, we suggest availing separate water sources for animal and human consumption, fencing all water sources to limit animal access, and using hand pump water sources over spring water for human consumption.

## Data Availability

To generate findings of this study, data was collected and analyzed based on the stated methods and materials. All the data were incorporated in the manuscript and no supplementary files accompanied the submission. The original data supporting this finding will be available at any time upon request.
